# Detecting flowering phenology in oil seed rape parcels with Sentinel-1 and -2 time series

**DOI:** 10.1016/j.rse.2020.111660

**Published:** 2020-03-15

**Authors:** Raphaël d’Andrimont, Matthieu Taymans, Guido Lemoine, Andrej Ceglar, Momchil Yordanov, Marijn van der Velde

**Affiliations:** European Commission, Joint Research Centre (JRC), Ispra, Italy

**Keywords:** Phenology, Rapeseed, Oil seed rape, Canola, Brassica napus, Copernicus, Monitoring, Sentinel-1, Sentinel-2, LUCAS, Crop modeling, Growing degree days, Crop yield forecasting, Climate change, Crop production, Anthesis

## Abstract

A novel methodology is proposed to robustly map oil seed rape (OSR) flowering phenology from time series generated from the Copernicus Sentinel-1 (S1) and Sentinel-2 (S2) sensors. The time series are averaged at parcel level, initially for a set of 229 reference parcels for which multiple phenological observations on OSR flowering have been collected from April 21 to May 19, 2018. The set of OSR parcels is extended to a regional sample of 32,355 OSR parcels derived from a regional S2 classification. The study area comprises the northern Brandenburg and Mecklenburg-Vorpommern (N) and the southern Bavaria (S) regions in Germany. A method was developed to automatically compute peak flowering at parcel level from the S2 time signature of the Normalized Difference Yellow Index (NDYI) and from the local minimum in S1 VV polarized backscattering coefficients. Peak flowering was determined at a temporal accuracy of 1 to 4 days. A systematic flowering delay of 1 day was observed in the S1 detection compared to S2. Peak flowering differed by 12 days between the N and S. Considerable local variation was observed in the N-S parcel-level flowering gradient. Additional in-situ phenology observations at 70 Deutscher Wetterdienst (DWD) stations confirm the spatial and temporal consistency between S1 and S2 signatures and flowering phenology across both regions. Conditions during flowering strongly determine OSR yield, therefore, the capacity to continuously characterize spatially the timing of key flowering dates across large areas is key. To illustrate this, expected flowering dates were simulated assuming a single OSR variety with a 425 growing degree days (GDD) requirement to reach flowering. This GDD requirement was calculated based on parcel-level peak flowering dates and temperatures accumulated from 25-km gridded meteorological data. The correlation between simulated and S2 observed peak flowering dates still equaled 0.84 and 0.54 for the N and S respectively. These Sentinel-based parcel-level flowering parameters can be combined with weather data to support in-season predictions of OSR yield, area, and production. Our approach identified the unique temporal signatures of S1 and S2 associated with OSR flowering and can now be applied to monitor OSR phenology for parcels across the globe.

## Highlights

• Novel Sentinel-1 & -2 based methodology for parcel mapping of OSR flowering phenology
• Peak flowering was detected with an accuracy of 1 to 4 days combining Sentinel-1 & -2.
• Mapping of 32,355 OSR parcels in Germany shows expected climatic gradient in flowering.
• Robustness confirmed by in-situ BBCH observations at parcels and phenology stations.
• Crop model-relevant thermal time to flowering based on satellite observations.

## 1 Introduction

During early spring each year, the bright yellow flowers of oil seed rape (OSR, *Brassica napus *L.**) appear across Europe, North America, China, India and Australia (Sulik and Long, 2015). OSR is a widely cultivated winter or spring oil seed crop. While aesthetically appealing to the eye, OSR is an intensively grown crop supplying vegetable oils, biodiesel, and animal feed (e.g. Van der Velde et al., 2009). Between 2000 and 2017, global OSR production has increased markedly (by a factor of 1.93) and OSR is currently the fourth crop by area in the European Union (EU). Production in the EU also almost doubled in less than 20 years from 11.3 million metric tons (MT, 10^3^ kg) in 2000 to 21.7 million MT in 2017 (Supplementary Fig. 1). This increase is largely driven by policy as successive EU directives raised the minimum percentage of biodiesel to be included in traffic fuels from 2% in 2005 (Directive 2003/30/EC), to then 5.75% in 2010 and to finally 10% in 2020 (Directive 2009/28/EC). Anticipating OSR production levels is thus crucial for food and energy markets.

Abiotic conditions during specific developmental stages strongly influence the crop's yield (Habekotté, 1993, Iglesias and Miralles, 2014, Tayo and Morgan, 1975, Tayo and Morgan, 1979, Zhang and Flottmann, 2018). For OSR, a critical period for yield information is the flowering or anthesis period (Kirkegaard et al., 2018, Zhang and Flottmann, 2018).The reduction of assimilation due to low solar radiation or water deficiencies during anthesis leads to limited sinks (i.e. less pods are formed) and higher abortion rates of flowers, pods, and seeds (Habekotté, 1993, Kirkegaard et al., 2018, Tayo and Morgan, 1979, Zhang and Flottmann, 2018), hence determining seed density produced, a parameter strongly related to yield (Diepenbrock, 2000, Iglesias and Miralles, 2014, Kirkegaard et al., 2018, Zhang and Flottmann, 2018). While there is a potential for yield compensation during the pod filling period (from 3% (Zhang and Flottmann, 2018) to 61% (Labra et al., 2017) increase in seed weight), low solar radiation and water stress during flowering reduced pods number twice as much as the same conditions during the pod development and filling stages (Zhang and Flottmann, 2018). Concurrently monitoring the OSR flowering period and meteorological conditions thus constitutes important information to assess expected production levels.

OSR flowering can be detected by optical remote sensing (Sulik and Long, 2015, Sulik and Long, 2016). Using optical data acquired during flowering presents challenges as the bright yellow flowers impact the most widely used spectral indices; NDVI for instance is decreasing during flowering (Shen et al., 2009, Sulik and Long, 2015). Due to cloud interference, incomplete visible and infrared time series may have limitations in consistently and accurately monitoring plant phenological changes. Therefore, Synthetic Aperture Radar (SAR) can be used to complement optical data (see e.g. Fieuzal et al., 2013). When OSR grows, the backscatter signal, the randomness in scattering, as well as the amount of scattering attributable to volume scattering increase (McNairn et al., 2018). The increase in biomass creates a thick and rough canopy structure as the leaves and pods from neighbouring plants often intertwine with one another (Cable et al., 2014), contributing to a strong back-scatter signal, in particular when canopy elements have characteristic sizes that are in the same order as the incident microwave wavelength (Tian et al., 2019). Flowering results in a temporary layer of smaller buds and flowers in the top canopy, which will modulate the increasing trend in backscattering.

Opportunities to combine optical and radar observations are provided by the EU Copernicus Sentinel-1 (S1) and Sentinel-2 (S2) satellite sensors, for which the generated data products are available under a full, free, and open license. In constellation, S1 and S2 have a nominal revisit capacity of respectively 6 days and 5 days, when combining the identical A and B platforms. However, since for S1 both ascending and descending orbit observations can be combined, the effective revisit of S1 is better than 6 days. Additionally, orbits are typically chosen to ensure seamless coverage of the sensor swath at the Equator. This causes swaths to overlap on the sides, especially towards higher northern and lower southern latitudes, thus effectively leading to a higher revisit in those overlaps (Lemoine, 2017). Data have a finest spatial resolution of 10 m which is ideal for monitoring crops at parcel level. Crop type mapping with Sentinels has already been demonstrated in various recent studies such as Belgiu and Csillik, 2018, Van Tricht et al., 2018, Immitzer et al., 2016, Defourny et al., 2019 using vegetation indices or backscattering. Similarly, several studies (e.g. Veloso et al., 2017, Vrieling et al., 2018) have shown that Sentinel data could be used for studying phenology.

Despite the proven capabilities of Sentinel satellites for crop mapping and estimating phenology, they have not yet been applied effectively for tracking crop development across large areas. Moreover, given that it should now be possible to monitor efficiently from single parcels to regions, scale-dependent information requirements can be satisfied relatively easily. Indeed, localized impacts at parcel level, e.g. late spring frost, may lower OSR yield potential dramatically, while the spatial extent of the frost and local growth progress of sensitive phenological stages (e.g. flowering) will determine the aggregate impact at regional scale.

### 1.1 Objectives

Improving spatial detail and better characterizing the timing of flowering through improved parcel-level monitoring should increase the accuracy of yield forecasting. In addition, the effects of the distinct flowering stage on the structural and spectral properties of the OSR canopy provide an opportunity to develop and test methods that rely on observations from various sources. In this study, we aim to demonstrate that it is possible to characterize critical OSR flowering parameters by combining the analysis of S1, S2, and in-situ data. The temporal accuracy and spatial continuity of these retrieved parameters are studied though specific research questions:

• Can we accurately detect the peak of OSR flowering with S1 and S2 time-series at parcel level?
• Are microwave S1 and optical S2 signals in agreement when characterizing peak flowering in OSR?
• How do the different revisiting frequencies of S1, S2 affect the temporal accuracy of the detection of peak of OSR flowering obtained from in-situ observations?
• Can the temporal trend and spatial pattern in detected peak flowering be related to a simple growing degree day (GDD) based phenology model?
• How can parcel level characterization benefit regional OSR monitoring and yield forecasting?

## 2 Materials and methods

The methodology is divided in 6 steps. The first section describes the study area and the *in-situ* data collection (Section 2.1). The second section describes how parcels are delineated both visually and by using classification methods on S2 imagery (Section 2.2). Subsequently, S1 and S2 time series are extracted and averaged on each parcel to derive the flowering peak (Section 2.3). The retrieved flowering phenology (Section 2.4) is then compared to the *in-situ* data and independent phenology observations by the German weather service – the Deutscher Wetterdienst (DWD) (Section 2.5). Finally, the method to accumulate temperature trough the growing season is described (Section 2.6).

### 2.1 In-situ data collection

#### 2.1.1 Survey: areas and timing

**Fig. 1:**
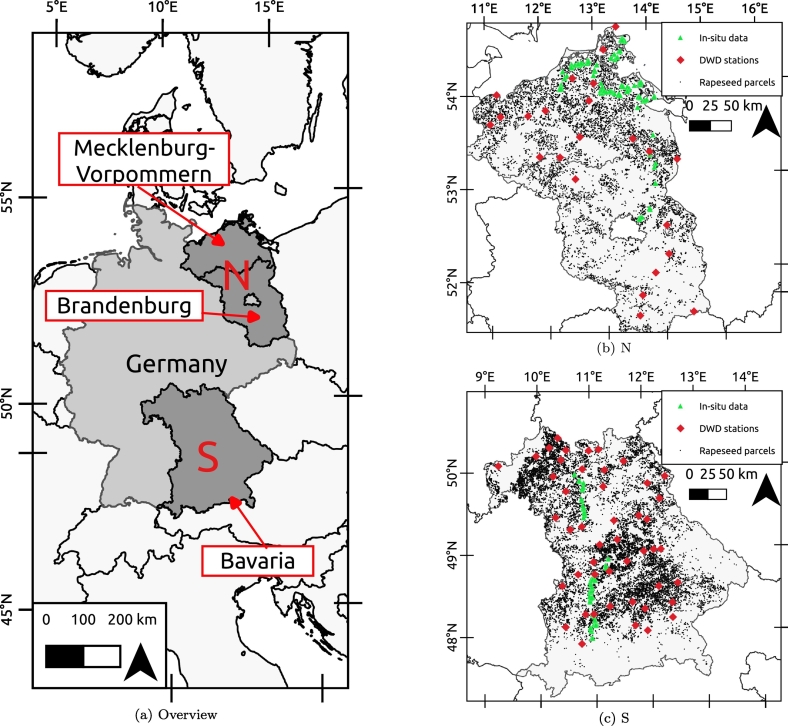
(a) Overview. The monitored regions in Germany. The northern region (N) consists of the federal states of Mecklenburg-Vorpommern and Brandenburg, and the southern region (S) is the federal state of Bavaria. (b) N. The northern region (N) contains 115 parcels for which in-situ data were collected; 23 DWD stations with phenology information; and 10,693 OSR parcels obtained by classification of S2. (c) S. The southern region (S) contains 114 parcels for which in-situ data were collected; 47 DWD stations with phenology information and 21,662 OSR parcels obtained by classification of S2. (For interpretation of the references to color in this figure, the reader is referred to the web version of this article.)

The German northern federal states Brandenburg and Mecklenburg-Vorpommern and the southern federal state of Bavaria were selected to monitor OSR flowering in this study (Fig. 1a). The advantage of these regions is that OSR is widely cultivated as a winter crop there, they lie along a climatic gradient, have a good road network to facilitate one-day surveys along long transects, and are covered by a network of independent phenology observations.

In the North (N), we covered a distance of 366 km (north-south gradient) and 191 km (east-west gradient). The N site covers 52,659 km^2^ with 5 main land cover (Supplementary Tab. 2): Non-irrigated arable land (43%), Coniferous forest (23%), Pastures (15%), Broad-leaved forest (6%) and Discontinuous urban fabric (4%).

In Bavaria (S), we covered a linear distance of 342 km between the most southerly and the most northerly parcel. The S site covers 70,345 km^2^ with 5 main land cover (Supplementary Tab. 2): Non-irrigated arable land (33%), Pastures (21%), Coniferous forest (21%), Mixed forest (9%) and Broad-leaved forest (5%).

The in-situ parcel survey data was collected during the 2018 OSR flowering period, each parcel was visited three times at one week interval to capture the gradient of flowering. An approximate route was set up based on the Germany Agricultural Landscape map, which provides a crop type map based on Copernicus Sentinel and Landsat observations (Griffiths et al., 2019). Using the web-based “Aktuelle Pflanzenentwicklung”-database of the Germany National Meteorological Service (Deutscher Wetterdienst, 2018), appropriate dates to start the field campaign were identified. To further guide the field survey, we also contacted experts from the Landesforschungsanstalt für Landwirtschaft und Fischerei Mecklenburg-Vorpommern (N) and the Bayerische Landesanstalt für Landwirtschaft (S), respectively, to retrieve an expert forecast for regional OSR flowering dates.

#### 2.1.2 In-situ photography and phenology labelling

For selected parcels along the survey route in which OSR was identified, at least one panorama picture and one close up picture of the OSR was taken on two corners of the parcel with a GPS-enabled digital camera (Sony DSC-HX400 V). These parcels were revisited on the two subsequent survey days. A total of 229 parcels was surveyed in detail (Table 1), 115 in the North (green triangles in Fig. 1b) and 114 parcels in the South (green triangles in Fig. 1c). Meta-data on date, time, and geographic location was included with the pictures.

**Table 1:**
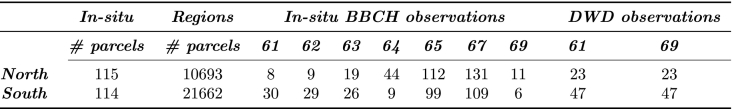
Field data collection summary.

**Fig. 2:**
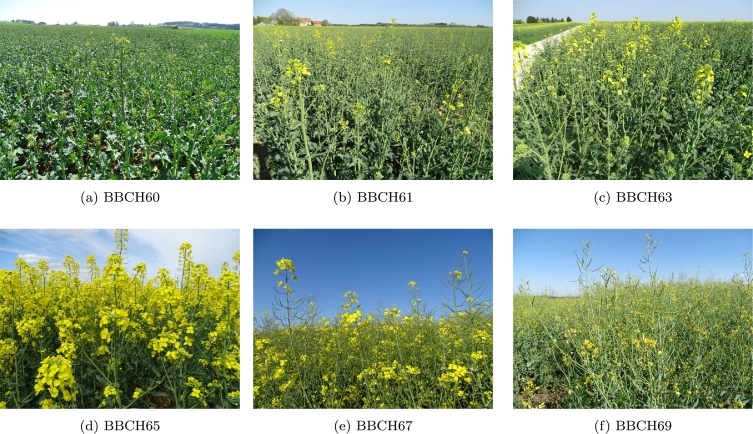
OSR in-situ images were photo-interpreted and classified according to the BBCH scale. The main flowering stages are presented here: (a) Buds are still closed and described as “yellow buds”, (b) up to 10 % of the flowers on the main raceme are open, (c) from 10 to 30% of the flowers on the main raceme are open, (d) full flowering, 50% of the flowers on the main raceme are open, (e) flowering is declining and most petals have fallen, (f) end of flowering. (For interpretation of the references to color in this figure legend, the reader is referred to the web version of this article.)

To determine phenological stage, the in-situ images were photo-interpreted per date for each parcel using the international Biologische Bundesanstalt, Bundessortenamt und CHemische Industrie (BBCH) scale (Lancashire et al., 1991, Meier, 2001, Weber and Bleiholder, 1990). The BBCH scale describes phenological stages of crops using criteria that link growth stage to a decimal code. The first digit indicates the principal stage of development (e.g. 6 = flowering) while the second digit relates to a secondary growth stage or to the percentage of plants in that stage. For OSR, the second digit is closely related to the progress of the flowering stage, with BBCH = 65 being considered the peak of flowering and BBCH = 69 the end of flowering (Meier, 2001). For each acquisition date, each parcel was thus classified according to the BBCH stages for flowering ranging from 61 to 69 (Fig. 2). The distribution of the parcels surveyed in the different BBCH stages is shown in Table 1. In the South, relatively more parcels were surveyed that were in the early stages of flowering. In the North, most parcels were already past these earlier stages when the survey started there. In summary, the peak of flowering in-situ data at parcel level contains 112 parcels in N and 99 parcels in S (Table 1).

### 2.2 Parcel delineation

As a pre-requisite to extract S1 and S2 time series, parcels with OSR need to be identified and delineated. The surveyed parcels were delineated by visual-interpretation (Section 2.2.1) while a classification method was applied to S2 imagery to retrieve large sets of OSR parcels in S and N (Section 2.2.2).

#### 2.2.1 Parcel delineation by visual-interpretation

Visual-interpretation of satellite images was used to manually delineate the 229 parcels for which in-situ data were collected. In order to delineate a OSR parcel, a S2 median composite centered on the flowering period. The S2 data composite time window ranges from May 1st to May 25th in N and from April 25th to May 10th in S. Parcel boundaries were manually delineated in QGIS by combining the composites with a Google Satellite layer from the Google satellite Tile Map Service, setting a transparency level of 40%, and the locations of the points where the in-situ images were collected.

#### 2.2.2 Parcel delineation with classification

To facilitate parcel delineations over the complete study area, a supervised classification on the S2 Level 1-C images was executed in Google Earth Engine (GEE, Gorelick et al., 2017). Training data for three different classes (i.e. OSR parcels, bare-ground, and other) were manually collected through photo interpretation of a median true color composite, using the GEE polygon drawing tool and extraction routines. A cloud-score algorithm was applied to the data before compositing to remove cloud-contaminated pixels. Copernicus Corine Land cover 2018 (European Environment Agency (EEA), 2018) was used to mask the non-agricultural classes to reduce the potential confusion with other land-cover classes, prior to applying the training.

A random forest classifier with ten trees was trained with the collected data for the North and South regions separately. A median temporal multispectral composite of cloud-free images available during the expected flowering period for both regions is used as input for classification: from May 1st to May 25th 2018 in the N and from April 25th to May 10th 2018 in the S. The S2 10 m resolution bands (B2, B3, B4, B8) together with the 20 m (B5, B6, B7, B8, B11 and B12) were used to create this composite. The 20-m bands were resampled at 10 m to obtain a 10-m composite.

The classifier is then applied to each of the regions on their respective composite. An erosion-dilatation kernel of radius 1 eroding three times and dilating twice is then applied to smooth the results and remove isolated pixel noise. The OSR class is exported as a set of polygons for which the geometry is simplified using a GEE built in simplification function and an error margin tolerance of 30 m. Finally, a filter is applied to the feature collection (i.e. all the polygons) to remove features smaller than 0.5 ha. This resulted in 10,693 and 21,662 parcels in the N and S respectively (Table 1). An example of the parcel photo-interpretation delineation and classification is illustrated in Supplementary Fig. 2. LUCAS micro survey data collected in 2018 (Eurostat, 2018) was used to validate the parcel delineation and rapeseed classification, i.e. 3895 LUCAS points in N and 4706 points in S. Supplementary Fig. 8 provides a map of the points used for the validation for N and S. The confusion matrices are shown in (see confusion matrices in Supplementary Tab. 1 a, overall accuracy and errors in Section 3.2).

### 2.3 Sentinel time-series extraction and OSR flowering signatures

#### 2.3.1 Sentinel-1

The S1 C-band operates at a central frequency (*ν*) of 5.404 GHz in the microwave portion of the electromagnetic spectrum which corresponds to a wavelength (*λ*) of 5.55 cm. S1 acquisitions over the European land mass are in the so-called interferometric wave (IW) mode, which registers the backscattering of a vertically transmitted microwave signal in a vertical and horizontal receiver, creating a VV and VH polarized band, respectively. Starting from the Ground Range Detected (GRD) Level-1 product, the data are pre-processed (in GEE) with the Sentinel-1 Toolbox to remove thermal noise and to create geocoded and radiometrically calibrated backscatter coefficients (*σ*^0^) at 10 m pixel spacing.

Since the leaves, stems and pods of OSR have sizes in the order of the C-band wavelength and form a dense structure of randomly oriented canopy elements, the crop tends to be an effective scatterer in VV. The smaller structured buds and flowers will create a temporary layer of less effective scatterers, which will partly shield the scattering of the underlying canopy, resulting in a drop in VV scattering as observed in Wiseman et al., 2014, Veloso et al., 2017, Vreugdenhil et al., 2018 and modeled by the cloud model (Attema and Ulaby, 1978). VH on the other hand, is more sensitive to the canopy depth and less sensitive to the change of canopy due to flowering. This is the reason why S1 $\sigma_{VV}^{0}$ were selected for the analysis. The values of the $\sigma_{VV}^{0}$ were extracted using GEE for every delineated parcel from January 1st to August 31st 2018.

#### 2.3.2 Sentinel-2

Before extracting the Sentinel-2 data, clouds and cirrus are removed using a cloud-score algorithm to mask contaminated pixels. Although atmospherically corrected S2 images are better for reliable spatial and temporal comparison, atmospheric correction is not always a prerequisite for classification and change detection (Song et al., 2001), especially when working with normalized indices. Therefore, the level-1C data are used in this study.

To capture the phenological development of OSR with optical remote sensing, the Normalized Difference Yellow Index or NDYI (Sulik and Long, 2016) (Eq. (1)) was selected as it should capture the increasing yellowness due to flowering.

(1) $$NDYI=\frac{\varrho_{Green}-\varrho_{Blue}}{\varrho_{Green}+\varrho_{Blue}}$$

The GEE code to extract average S1 and S2 along with the S2 classification is available on https://github.com/rdandrimont/rapeseed.

### 2.4 Peak flowering detection

Importantly, when characterizing the date of peak flowering one has to consider the revisit frequency of the survey, of the Sentinel Satellites, as well as the fact that peak flowering in OSR can last for several days. To accurately detect the timing of peak flowering at parcel level, three steps are needed. First, a method is developed to automatically detect the date of peak flowering (i.e. BBCH65) from smoothed S1 and S2 time-series (Section 2.4.1). Second, the peaks are retrieved and the outliers are removed (Section 2.4.2). Third, the consistency between S1 and S2 peak flowering detection is investigated (Section 2.4.3) and compared following a decomposition of systematic and unsystematic deviations.

#### 2.4.1 Peak flowering from smoothed satellite time series extreme

To find the peak flowering date at parcel level, an automated method was developed that computes the extremes of smoothed Sentinel time-series. The extremes are computed to find the dip (local minimum) and peak (local maximum) in the smoothed S1 and S2 time-series respectively. Although the choice of smoothing methodology could lead to uncertainty in the retrieval of phenology (Helman, 2018), the revisited Whittaker smoother (Atzberger and Eilers, 2011a, Eilers, 2003) was selected as it is easily implemented and does not need to meet any *a priori* assumption and as it was demonstrated to be one of the best smoother for remote sensing time series by Geng et al. (2014).

The revisited Whittaker filter (Eilers, 2003) aims at fitting a smooth series *z* to a noisy series *y*. As explained by Atzberger and Eilers (2011b), it does so while balancing two conflicting goals: the fidelity to the data *S*; and *R* the roughness of *z*. The sum *Q* (Eq. (2)) balances out the two goals. It combines a measure of the lack of fit to the data, given by (Eq. (3)) as a sum of squared difference with the roughness of the smoothed curved *R* expressed by second order differences (Eq. (4)). A series *z* is thus found that minimizes Q. The factor *λ* is the smoothing parameter. Increasing the *λ* strengthens the influence of *R* on *Q*, generating a smoother *z* at the cost of the degradation of the fit.

(2) Q=S+λR

with

(3) $$S=\underset{i}{\sum }{\left(y_{i}-z_{i}\right)}^{2}$$

(4) $$R=\underset{i}{\sum }{\left((z_{i}-z_{i-1})-(z_{i-1}-z_{i-2})\right)}^{2}$$

The value of the Whittaker smoothing parameter was obtained by cross validation (Atzberger and Eilers, 2011b). By leaving out each of the non-missing elements of *y* in turn, it is possible to smooth the remaining data and to predict the *y*_−*i*_ for each *y*_*i*_ left out. This hence allows for the computation of a cross-validation standard error (Eq. (5)), where *m* corresponds to the length of the series *y*.

(5) $$S_{cve}=\sqrt{\underset{i}{\sum }\frac{{\left(y_{i}-y_{-i}\right)}^{2}}{m}}$$

The cross-validation standard error *S*_*cve*_ is computed for the time series of each parcel and averaged for both satellites on all the parcels for which in situ data is available in both regions. This provides a mean error associated to each *λ* values. The optimisation is then undertaken to find the value of *λ* that minimizes *S*_*cve*_. The mean error was computed for values of *λ* ranging from 1 to 100, applied to both S1 and S2, that correspond to the degree of smoothness that was deemed appropriate through visual appreciation of the smoothed curve. The smoother and the cross-validation metric were implemented in R (R Core Team et al., 2013). The values of *λ* that minimize the cross-validation standard error of fitting S1 and S2 time series were respectively 1 and 2.

**Fig. 3:**
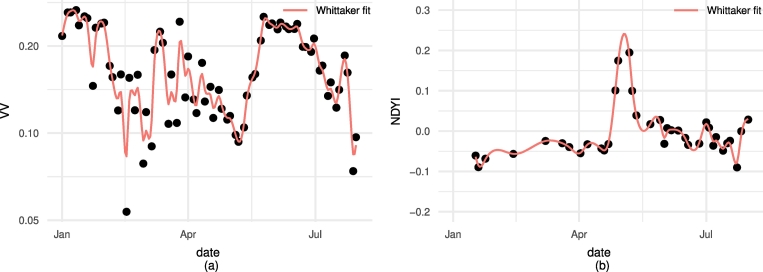
Example of time series obtained for a large parcel in the South of (a) $\sigma_{VV}^{0}$ and of (b) NDYI as smoothed with the Whittaker method.

The extraction provided smoothed S1 $\sigma_{VV}^{0}$ and S2 NDYI temporal profiles for each individual parcel as illustrated in Fig. 3.

The local maximum and minimum are then obtained to retrieve respectively the date of peak NDYI and of the minimum of VV. Those extremes are thus computed for all parcel averaged S1 and S2 time-series. If more than one extreme is obtained, only the peak or drop corresponding respectively to the highest value of NDYI or the lowest value of VV within the expected flowering period (i.e. April 21st to May 30th) is kept.

#### 2.4.2 Parcel signatures and peak flowering detection

Whereas the 229 parcels with in-situ data were visually delineated, the 32,355 parcels obtained by classification may contain errors. As many parcels are available, the classified parcels with a NDYI flowering peak below the 10th percentile are removed. This corresponds to removing the time series which have a maximum NDYI value within the expected flowering period of less than 0.0715 in the North, corresponding to 10th percentile, and of less than 0.078 in the South region.

First, the S1 and S2 derived flowering peak for the surveyed parcels are evaluated against the in-situ observations. In a second step, these S1 and S2 peak flowering dates are then averaged for the 115 and 114 surveyed parcels in N and S respectively. Thirdly, the S1 and S2 peak flowering dates are extracted for all of the parcels derived from the S2 classification.

Finally, the peak flowering date is computed for all parcels in both regions to display the flowering gradient in the North and in the South.

#### 2.4.3 Agreement S1 and S2

To assess the agreement between S1 and S2 across scales, the detected peak flowering dates are evaluated for parcels with in-situ observations and for all parcels around DWD stations. The agreement is assessed by characterizing the linear relationship between the dates computed from both time-series (S1 = X and S2 = Y). The Pearson correlation coefficient *r* is first computed. Second, a symmetric index of agreement, *L* (Eq. (6)), developed by Duveiller et al. (2016) is used to assess the degree of agreement of the peak flowering dates computed by both Sentinel time-series.

(6) $$L=\frac{2}{\sigma_{X}/\sigma_{Y}+\sigma_{Y}/\sigma_{X}+{\left(\bar{X}-\bar{Y}\right)}^{2}/(\sigma_{X}\sigma_{Y})}.r$$

Third, the deviation from agreement between the two sets of dates is decomposed in systematic and unsystematic deviation (Duveiller et al., 2016). This is done using an eigen decomposition of the covariance matrix of X and Y, yielding a line corresponding to the principal axis of the X-Y space and a vector *h* of the orthogonal distances between all X-Y points and this axis. The mean squared deviation can then be computed from the distance vectors and corresponds to the unsystematic component of the deviation *δ*_*u*_. The total deviation *δ* of the data from agreement corresponds to the mean squared deviation of the data cloud to the identity axis. According to Eqs. ((7), (8)) the ratio of the systematic deviation component *δ*_*s*_ and the total deviation *δ* yields the proportion of systematic deviation or bias.

(7) $$\delta =\delta_{s}+\delta_{u}$$

(8) $$f_{sys}=\frac{\delta_{s}}{\delta }$$

In addition, the relative accuracy and precision of the peak date detection is investigated for each of the Sentinel time-series. The dates corresponding to the S1 dip and S2 peak are compared to the date at which the BBCH65 stage (i.e. peak flowering) was observed in the field. The difference in days between the computed flowering peak date and the in-situ observation is calculated for each parcel. When two consecutive in-situ observations of one parcel correspond to peak flowering, and the time-series derived peak date falls between those two dates the difference is considered to be zero. The distribution of the days of difference between the BBCH65 as observed in-situ and as detected remotely is discussed in the context of location and observations made and sensors used.

### 2.5 Comparison with phenology observations

For the surveyed parcels, the flowering signature and the temporal detection accuracy of peak flowering as detected with S1 and S2 is compared with the in-situ BBCH information. To compare the flowering signature for the larger sets of parcels without in-situ data, independent reference data was obtained from the Deutsche Wetterdienst (DWD) phenology monitoring network (Kaspar et al., 2015) for the available stations represented in red on Fig. 1b and c). The DWD provides in-situ observations of the crop phenological stages following the BBCH scale (Meier, 1997) throughout Germany. For OSR, the dates of onset (i.e. BBCH61) and end of flowering (i.e. BBCH69) are available - but peak flowering is not (i.e. BBCH65). Each observation associated to a DWD station is considered representative of the crops that fall within a maximum distance of 5 km of this location. For our study, observations at 23 stations in the N and at 47 stations in the S are available (Table 1).

### 2.6 Growing degree days

Crop phenological development is strongly related to the accumulation of heat or temperature units above a threshold or base temperature below which little growth occurs. Although temperature is the most important factor controlling phenological development of OSR, it does so in combination with other factors such as vernalization, photoperiod, and water availability (Böttcher et al., 2016). To properly account for these factors within the phenological model, the information on sowing and/or emergence dates together with vernalization requirements would be necessary (Ceglar et al., 2019). As we do not have this information, we apply here a simpler Growing Degree Days (GDD) model that starts to accumulate heat after 1 January to estimate crop development. The GDDs are calculated by subtracting the crop's lower base temperature from the average daily air temperature:

(9) $$GDD=\overset{}{\underset{days}{\sum }}\left(\frac{\left(T_{\max}+T_{\min}\right)}{2}-T_{base}\right)$$

where 1 January is used as a starting date. In OSR, the lower base temperature (*T*_*base*_) is determined as being 3 °C as in Marshall and Squire, 1996, Böttcher et al., 2016. If the daily average temperature is below 3 °C, than the *GDD* value for that day is 0. Observed daily minimum and maximum air temperatures were obtained from the MARS Crop Yield Forecasting System (MCYFS) database, established and maintained by the Joint Research Centre of the European Commission for the purpose of crop growth monitoring and forecasting (Biavetti et al. (2014), https://agri4cast.jrc.ec.europa.eu/DataPortal/). Following a distribution of peak flowering dates for the parcels within a given grid cell, *GDD* is calculated for the *q*10, median, and *q*90 flowering dates.

## 3 Results

The in-situ determined BBCH stages are compared to the Sentinels' temporal profiles averaged over the 229 parcels to illustrate how the signatures correspond to the specific flowering phenology stages (Section 3.1). The satellite-retrieved peak date is then compared to in-situ data. Second, Section 3.2 assesses the temporal consistency of S1 and S2 in detecting the flowering peak for the parcels with in-situ data and for the parcels obtained by classification. The distribution of flowering peak dates obtained by satellite is compared to the DWD observed start and end of flowering (Section 3.3). Finally, the relation between satellite flowering phenology and GDD data are assessed in Section 3.4.

### 3.1 Satellite time series flowering patterns compared with in-situ data

**Fig. 4:**
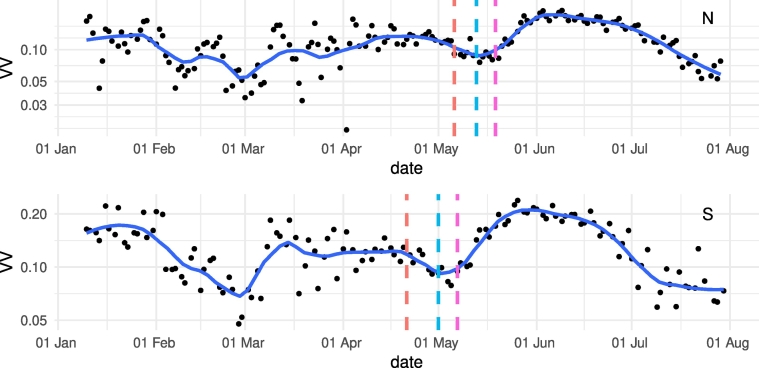
Spatially averaged S1 $\sigma_{VV}^{0}$ time-series for all N and S parcels compared with median dates of onset, peak, and end of flowering provided by in-situ observations. The dashed red line corresponds to the median date of onset (BBCH61), the dashed blue line to the median peak flowering date (BBCH65), and the dashed pink line to the median date of the end of flowering (BBCH69). (For interpretation of the references to color in this figure legend, the reader is referred to the web version of this article.)

**Fig. 5:**
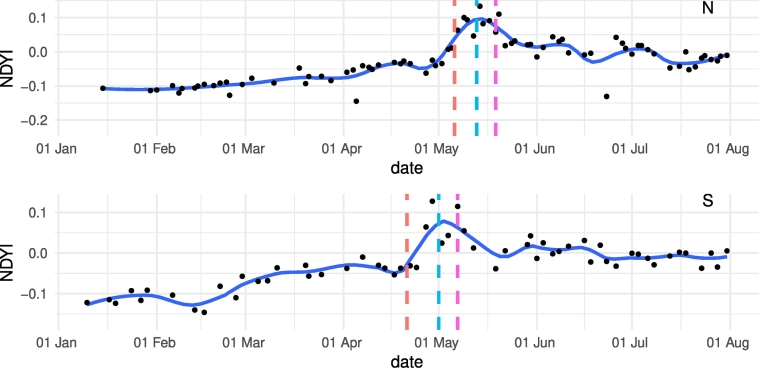
Spatially averaged S2 NDYI time-series for all N and S parcels compared with median dates of onset, peak and end of flowering provided by in-situ observations. The dashed red line corresponds to the median date of onset (BBCH61), the dashed blue line to the median peak flowering date (BBCH65), and the dashed pink line to the median date of the end of flowering (BBCH69). (For interpretation of the references to color in this figure legend, the reader is referred to the web version of this article.)

The BBCH stage for each of the 229 manually delineated parcels (green triangles in Fig. 1b and c) is determined for the three survey dates. The median date for the BBCH flowering stages (Table 2) are compared with the parcel-averaged S1 and S2 signatures (respectively Fig. 4, Fig. 5).

**Table 2:** Median date of occurrence of flowering stages obtained through photo-interpretation of the close up and panoramic pictures for the 229 parcels in N and S.

BBCH stage	N	S
BBCH61 (early flowering)	06-05-2018	21-04-2018
BBCH65 (full flowering)	13-05-2018	01-05-2018
BBCH69 (end of flowering)	19-05-2018	07-05-2018

The S1 $\sigma_{VV}^{0}$ time series are shown in Fig. 4. The time series are averaged for 115 parcels in the North and 114 parcels in the South. The in-situ determined peak of flowering corresponds to the local minimum in the S1 $\sigma_{VV}^{0}$ time-series. The onset and end of flowering are respectively related to the downward and upward curvature marking the convex dip. The onset and end of flowering is not exactly marked by a detectable feature of the time-series since no clear inflection point, minimum, or maximum is observed. Nevertheless, the in-situ data is in line with the S1 signal.

Similarly to S1, S2 NDYI time series were extracted and averaged for the two sets of regional parcels (Fig. 5). The in-situ measured peak of flowering date corresponds to the averaged peak of NDYI while the onset of flowering in the South corresponds to the onset of the NDYI peak. The lines do not clearly align with detectable features in the NDYI signal for the onset in the North and the end of flowering in N and S.

The flowering signature as characterized by the S1 $\sigma_{VV}^{0}$ and the S2 NDYI time-series is consistent for both sensors and in both areas considered (N and S, Fig. 4, Fig. 5). Moreover, the North to South flowering gradient is consistently captured by the remotely-sensed observations. On average, flowering occurred 12 days later in the North compared to the South (see Supplementary Fig. 4).

**Fig. 6:**
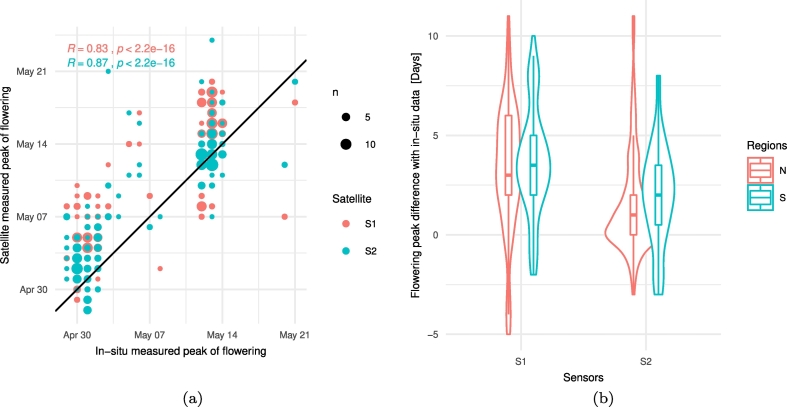
Comparison between detection of peak flowering date for each parcel from S1 and S2 time-series with in-situ observation (N = 229). (a) Scatter plot and correlations between satellite and in-situ observations (S1 R = 0.83; S2 R = 0.87): (b) Distribution of the difference in days between the peak flowering date detected by S1 and S2 and by in-situ observed peak flowering for both regions.

The peak of flowering date was extracted for each of the 229 parcels from S1 and S2 and compared to in-situ observations (Fig. 6). The correlation between the satellite-retrieved peak of flowering and the in-situ observation is 0.83 for S1 and 0.87 for S2 (Fig. 6a). The difference in time (days) between S1 and S2 detected peak flowering date and the in-situ determined date is shown in Fig. 6b. The median difference for S1 (Fig. 6b) is 3 days for parcels in both the N and S, while for S2 it is 1 day for the northern parcels to 2 days for the southern parcels. Furthermore, for 50*%* of the parcels in the South, the S1 detected peak is delayed by 2 to 5 days while this delay is 1 to 3.5 days for S2. In the North, this delay ranges between 2 to 6 days for S1 and from 0 to 2 days for the S2 detected peak. In conclusion, S1 and S2 capture the peaks of flowering with a median delay below 5 days.

### 3.2 Parcel level peak flowering detection and scale comparison

The classification resulted in 32,355 OSR parcels that were validated with 2018 LUCAS OSR observations (See Supplementary (confusion matrices and accuracy metrics in Supplementary Tab. 1). The overall accuracies range from 0.9220 (N) to 0.9894 (S), the OSR commission errors from 4.02% (N) to 16.67% (S) and the OSR omission errors from 5.49% (N) to 36.27% (S). In addition, the parcels' geometries were compared with the photo-interpreted parcels indicating on average less than 5% of area difference (Supplementary Fig. 3).

**Fig. 7:**
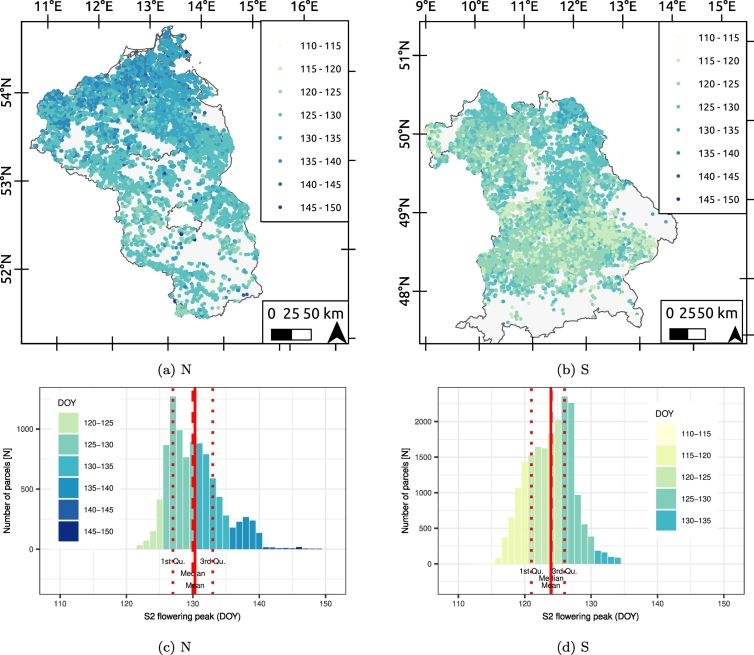
Flowering peak (Day Of Year; DOY) as obtained by S2 over the parcels obtained with classification (N = 32,355).

The peak flowering date as observed by satellite is determined by computing the extremes of the S1 and S2 profiles of the parcels obtained by classification. Fig. 7 displays the Day of Year (DOY) of S2 detected peak of flowering at parcel level for both regions confirming the South to North flowering gradient (Fig. 7a for N and Fig. 7b for S). The distribution of the mapped points are shown as histograms in Fig. 7c for N and Fig. 7d for S.

**Fig. 8:**
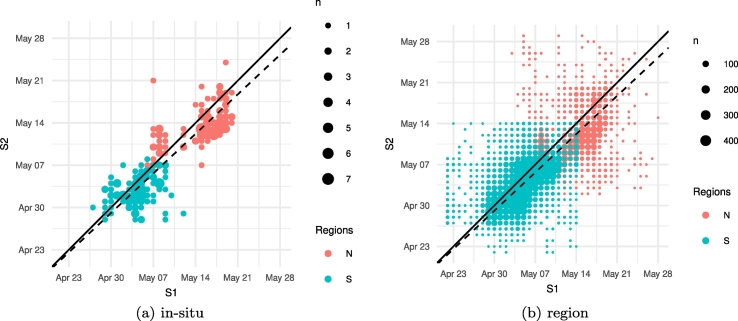
Comparison between detection of peak flowering date for each parcel from S1 and S2 time-series for (a) parcels with in-situ data (N = 229) and for (b) all classified parcels (N = 32,355). The identity line is solid while the dashed line corresponds to the principal axis of the X-Y data.

The S1 and S2 determined parcel level peak flowering dates are compared in Fig. 8. The correlation between the two datasets along with the value of *L* and the proportion of systematic deviation are presented in Table 3. The consistency in S1 and S2 peak detection is displayed for the 229 parcels with in-situ data (Fig. 8a) and for the 32,355 parcels obtained by classification (Fig. 8b).

**Table 3:** Pearson's correlation, symmetric agreement index and proportion of systematic deviation values calculated between the peak flowering dates obtained from S1 and from S2 time-series.

Parcels	*r*	*L*	*f*_*sys*_
Parcels with in-situ data (N = 229)	0.8605	0.8350	0.1807
Parcels without in-situ data (N = 32,355)	0.7086	0.6840	0.1109

The comparison between S1 and S2 derived peak flowering dates for parcels with in-situ data (Fig. 8a) shows a systematic tendency of S1 to detect the peak of flowering later than S2. In addition, the computed metrics show that there is a strong linear relationship between S1 and S2 detected peaks and that 18.07*%* of the deviation observed is due to a systematic bias (Table 3). The symmetric index of agreement moreover shows that overall there is no important bias between the peak detection from the two sensors at this scale.

Comparing the detected peak from both Sentinels for the parcels without in-situ data shows a consistent relationship in the way both sensors detect flowering (Fig. 8b). Nevertheless, in this case the systematic agreement index shows that a higher proportion of the deviation is due to bias (Table 3). The systematic bias remains relatively low while the unsystematic bias constitutes an important component of the deviation (i.e. > 88*%* of the bias). This means that the observed bias is mainly due to noise. Moreover, the S1-identified VV backscatter minimum is delayed with respect to the observed peak flowering.

### 3.3 Comparison with Deutscher Wetterdienst phenology data

**Fig. 9:**
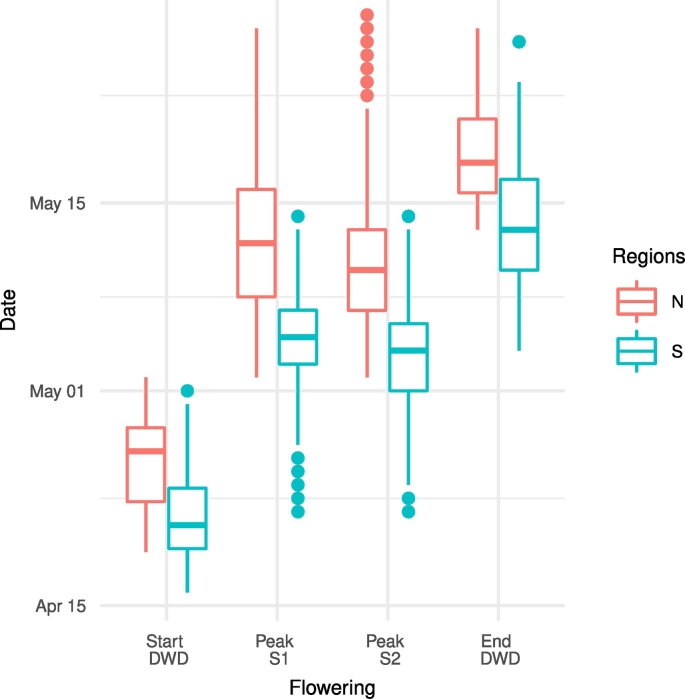
BBCH start and end of flowering observed by the DWD network and peak flowering as detected by S1 and S2 for each of the regions.

Peak of flowering information is not available from DWD, and, as the peak of flowering is retrieved from satellite, a strict validation is not possible. However, Fig. 9 indicates the range of start and end of flowering as obtained from DWD data along with the peak obtained by the Sentinels over both regions. The flowering peak dates obtained from S1 and S2 are between the start and end observed at the DWD stations in N and S.

### 3.4 Growing degree days to reach flowering

**Fig. 10:**
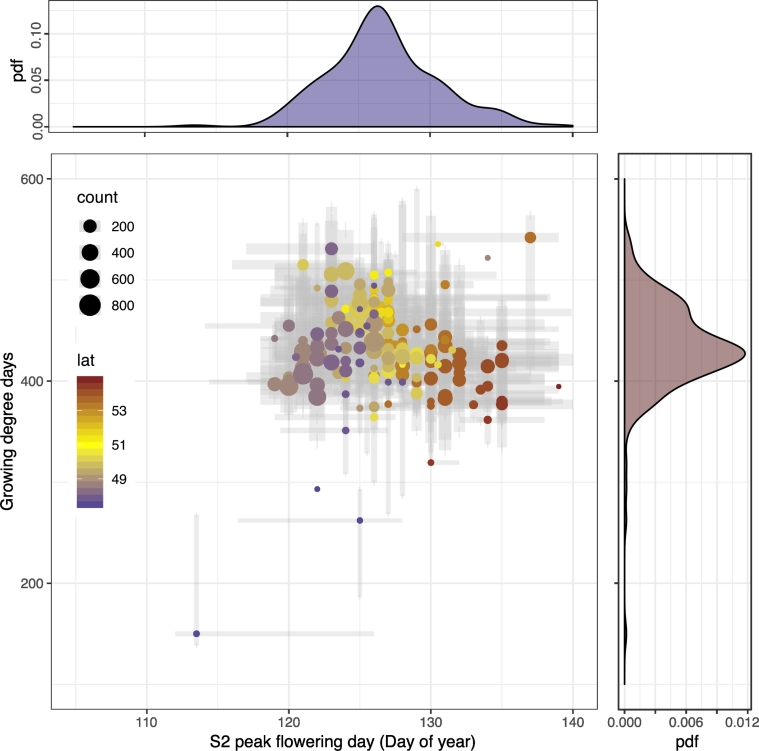
Comparison of flowering peaks DOY obtained by S2 and GDD at 25-km grid. The horizontal bars represent uncertainty related to observed day of flowering within grid cells while the vertical bars represent uncertainty related to GDD estimation based on different parcel observations from each grid cell. The color of bubbles in the scatterplot reflects the latitude of location, where parcels are located. Bubble size indicates the number of parcels per each grid point taken into consideration. Marginal probability densities of peak flowering days and growing degree days are shown on top and right of the main plot, respectively. The correlation between S2 flowering date and the latitude is higher (*R* = 0.81,*p* < 0.001) than the correlation between S2 flowering date and GDD (*R* = −0.04,*p* = 0.52). (For interpretation of the references to color in this figure legend, the reader is referred to the web version of this article.)

The GDD needed to reach peak flowering was calculated with temperature data from the MARS 25-km grid accumulated since January 1, 2018. Based on the distribution of peak flowering dates for all parcels within a given grid cell, GDD is calculated for the 10 percentile, median, and 90 percentile. The 25-km grid cells contain on average 148 parcels (from 1 to 812, see Supplementary Fig. 6 (a) for the spatial distribution and 6 (b) for the histogram). Fig. 10 shows the relation between peak flowering ranges and the corresponding accumulated GDD. Bubble size indicates number of parcels. The probability density functions indicate a range of flowering and GDD values that center around DOY 123 and a GDD of 425 °C. Changes in detected flowering date are not related to GDD as expected, different parcels reach flowering according to sowing date and local thermal accumulation (*R* = −0.04,*p* = 0.52). On the contrary a clear latitudinal trend (*R* = 0.81,*p* < 0.001) is depicted in the data as a consequence of climate variability and agricultural practices.

**Fig. 11:**
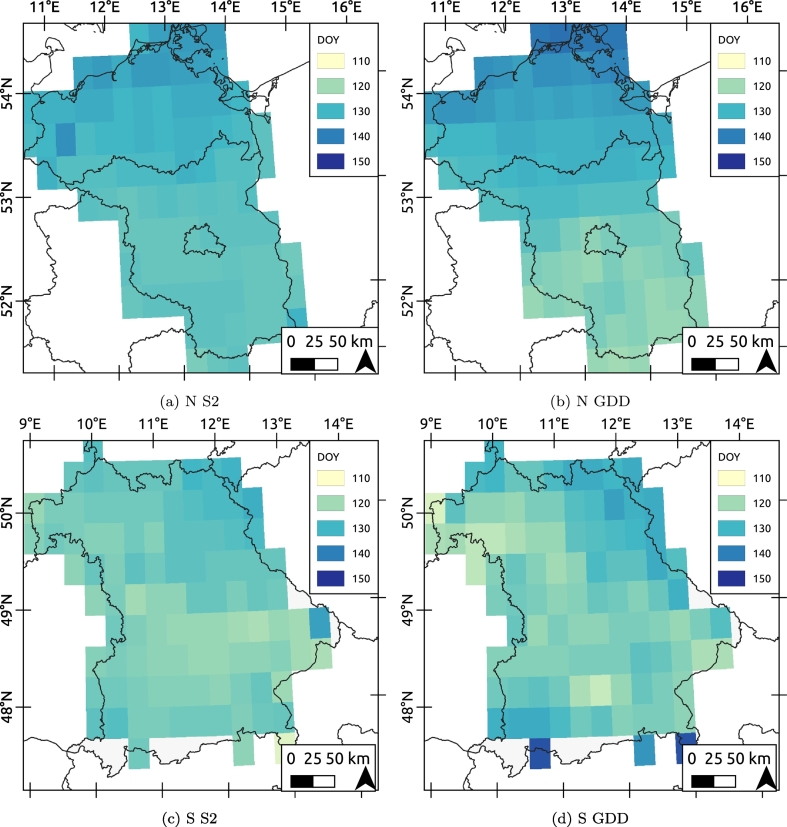
Median flowering peak (Day Of Year; DOY) as obtained by S2 aggregated on a 25-km grid in N (panel a) and S (panel c). DOY to reach a GDD sum of 425 in N (panel b) and S (panel d).

Gridded crop models are used to provide predictors for statistical forecasts such as in the MARS Crop Yield Forecasting System (MCYFS, e.g. Van der Velde et al. (2018)). Increasingly, gridded crop models are also used in e.g. agri-environment assessments evaluating resources use efficiency, and future climate adaptation and mitigation potentials (e.g. Balkovič et al., 2018). To bridge the scale from parcel to a typical 25-km gridded crop model, we evaluate which date of flowering would be obtained if we had simulated a OSR variety with a 425 GGD requirement to reach flowering in the regions of interest (Fig. 11). In this way, we provide a template as to how such valuable in-season parcel level information may be assimilated in a crop model used for e.g. in-season forecasting.

The resulting maps (Fig. 11) display the median parcel-level flowering date observed by S2 in the N (Fig. 11a) and S (Fig. 11c) and the calculated flowering date given a heat requirement of 425 GDD on a 25-km grid in N Fig. 11b) and S (Fig. 11d). Overall, the correlation (*r*^2^) between the GDD calculated flowering dates and S2 median observed flowering date in the 25-km grid cells for the N and S respectively are 0.84 and 0.54. Moran's *I* (Anselin, 2005) was calculated to asses global spatial autocorrelation on the 25-km grid. Moran's *I* is 0.34 for the South and 0.63 for the North (see Supplementary file Fig. 5). This indicates that flowering dates were more strongly clustered in the North.

## 4 Discussion

The peak of flowering is consistently captured by both S1 and S2 observations in both areas. The S1 $\sigma_{VV}^{0}$ backscattering coefficient is characterized by a pronounced drop during flowering (Fig. 4). In the first part of the growing season, before canopy closure, the signal is more noisy. This is likely related to surface conditions (e.g. rain, snow, frost) when the parcels are only partially covered with emerging OSR. Furthermore, this is exaggerated by not having separated ascending and descending orbits for S1, so that cultivation direction may play a significant role in backscattering of partially covered parcels. As soon as the crop closes this variability disappears (i.e. early to mid-April) confirming the isotropic behavior in backscattering of the OSR canopy. During the OSR flowering period, S1 detection is less sensitive to the SAR look direction, so that ascending and descending orbit observations can be combined, leading to denser time series.

The NDYI index captures the increasing yellowness in the green waveband as a signature in the index time-series (Fig. 5). The yellowness of OSR petals is due to their content in carotenoid pigments absorbing wavelengths of ~450 nm (Sulik and Long, 2016). By absorbing the blue light they reflect a mix of green and red light that we perceive as yellow. When flowering occurs the leaves transfer their nutrients to new branches, buds, and flower racemes (Tayo and Morgan, 1975), hence reducing the photosynthetic activity of the plant and its associated reflectivity. In turn, when reaching the end of the flowering period the biomass is allocated primarily to the newly created pods (Brunel-Muguet et al., 2013). Note how the NDYI stays at a higher overall value after flowering than before flowering. This confirms the growth of a reproductive canopy layer that induces a different appearance in S2, followed after the peak, by the senescence process. Decreasing chlorophyll content at senescence induces reduced absorption in the red and comparatively higher carotenoid content leading to relatively higher absorption of blue light (Sulik and Long, 2015, Tańska et al., 2017).

Comparing the satellite-detected peak with the in-situ data from the 229 parcels indicates a delay less than 5 days (Fig. 6b) which is below the nominal revisit capacity of the sensors. A deeper consistency analysis between S1 and S2 reveals that S1 detects flowering peak with a systematic delay. A likely explanation is that the attenuating effect on the C-band VV backscattering of the flowering layer has a maximum shortly after peak flowering stage BBCH50. The in-situ data collection was carried out once a week limiting our knowledge of the exact moment of peak flowering within a 7-day period. Additionally, the photo-interpretation process could lead to some errors. Future studies should target a more frequent revisit close to the flowering stage. Alternatively the use of crowdsourced street-level pictures could be considered as source of in-situ data (d’Andrimont et al., 2018b).

The second comparison of the flowering peak obtained by S1 and S2 with the 70 in-situ observation stations of the DWD shows that the S1 and S2 derived flowering signal was consistent, i.e. that the flowering peak date is between the start and the end of flowering measured at the stations. The DWD observations do not report the flowering peak stage and the S1 and S2 time series do not exhibit strong features at start and end of flowering, which limits a direct comparison.

This study demonstrated the strengths and the consistency of the S1 and S2 constellations to monitor flowering stages of OSR at the parcel level. The reliability of peak flowering detection is not significantly different between S1 and S2, apart from an approximate 1.5 day delay in the date determination. The proposed methodology was demonstrated over two large and contrasting German regions. The methodology could also be applied across even larger extent to e.g. characterize the full agro-phenological gradient across Europe. However, one limitation of the method is the need to delineate OSR parcels. In the current study, the OSR parcels were obtained with a supervised classification that required the collection of training data. This collection is time consuming and reduces the method's automation potential. Several approaches could mitigate this limitation. For example, a novel mapping method tailored to map OSR automatically with S2 was recently proposed by Ashourloo et al. (2019). Similarly, existing administrative crop type maps could be used, such as the Crop Data Layer (Boryan et al., 2011) in the U.S. or the European Member States' Land Parcel Identification Systems (LPIS), which provide detailed digital geometries of agricultural reference parcels. A third approach could be to use generic continental crop type maps obtained from remote sensing such as proposed by Massey et al. (2018) for North America and by Teluguntla et al. (2018) for China and Australia.

The frequent revisits and rapid processing of the S1 and S2 data makes the phenology detection particularly suited for in-season crop forecasting. Although the spatial and temporal accuracy of the information in our example (Fig. 10) had purposefully been degraded, such assumptions provide a work-flow for e.g. implementation in operational in-season crop forecasting. For instance, flowering phenology dates could dynamically update crop growth models running in real-time, or could be used to develop statistical models relying on spatially precise information on flowering onset, peak, and length, and e.g. temperature data.

Due to dry weather in 2018 (Buras et al., 2019, Toreti et al., 2019), conditions were particularly suited for observations in the visible spectral range, which has benefited the cross-comparison between S1 and S2 time series. These particularly dry and hot conditions also explain the relatively short and advanced growing stages in the North. Long term weather records for both N and S regions show that extended periods of cloudy weather are more probable in the spring flowering period of OSR which suggest that using S1 time series can be opportune in such years.

As stated in the introduction, the flowering period is critical for yield formation (Kirkegaard et al., 2018, Zhang and Flottmann, 2018), as are the temperature and water stress conditions during this period. OSR has only limited possibilities to compensate for adverse conditions during flowering, since oil concentration of the harvested product primarily depends on the seed filling period after flowering. While this study proposes a method to detect peak of flowering, the timing, the abundance and the length of flowering have to be considered for yield estimation. Additionally, sowing date, crop density and homogeneity, as well as genetic characteristics of the cultivars and fertilization timing constitute other important factors (Diepenbrock, 2000). These parameters should be considered for further studies.

The 2018 heat shortened the length of flowering in affected parcels. We also observe a short time period (i.e. <4 days; see Supplementary Fig. 7) between flowering peaks of the different parcels within a grid cell. This seems short with respect to a year with normal weather conditions given the variety of sowing dates, micro-climates, cultivar selection and agro-management practices affecting the 100s of parcels within a 25-km grid cell. Indeed, the spatial pattern seems to reflect those areas most affected by heat. Crop modeling could further elucidate the drought and heat effects.

In the context of large scale gridded crop modeling, data on flowering phenology, especially if available for a number of years, would provide very valuable and comparatively spatially detailed information. Ideally, the full distribution of the parcel level GDDs determined within a grid cell should be used to properly model and represent real parcels. Alternatively, crop models may operate on a sufficient number of parcels for which detailed meteorological, soil, and crop management information is available.

The findings in this study are related to autumn sown OSR. Apart from intra-parcel variation in OSR varieties, the actual sowing dates in autumn, which can range from early September to mid-October, may lead to significant differences in crop development before hibernation, depending on regional weather conditions. These differences, and the impact of local winter temperatures and snow conditions, contribute to the local and regional variation in flowering phenology. The presented GDD based method does not take such differences into account, as GDD is accumulated only after January 1, 2018, possibly leading to a lower spatial consistency in observed and modeled patterns.

Finally, accurately characterizing the seasonal radiometric signatures of crops is also directly relevant for agricultural policy. To monitor agricultural activities on agricultural land receiving income support under the Common Agricultural Policy in the EU, the so-called ‘ marker’ concept was introduced (Devos et al., 2017). This refers to observing temporal S1 and S2 signatures that are specific to agricultural activities performed on the parcel or to crops and their development as done in this study. For example, detecting crop type, or the dates when parcels are ploughed, mown and harvested. Currently, a few studies elaborate on this concept, with a focus on non-permanent grassland (d’Andrimont et al., 2018a, Kanjir et al., 2018). In future work, detailed investigations in the use of S1 and S2 signatures to detect other crop phenology features and agricultural activities should be carried out. Further research could focus on determining the elongation stage in cereals, the green up of sugar beet and potatoes, the flooding of rice, as well as determining the senescence and harvesting periods of various crops.

## 5 Conclusions

S1 and S2 (provided enough cloud free acquisitions are available), can detect peak flowering of OSR at parcel level with a temporal accuracy of 1–4 days. The S2 signature is more pronounced whereas detection by S1 is slightly delayed with respect to S2. Importantly, S1 allows flowering detection in cloudy conditions. Collected in-situ data and DWD phenology observations confirm that onset and end of flowering relate to the downward and upward curvature marking the convex dip in S1 backscattering and the increase and decrease in peak NDYI as detected by S2. Along an overall parcel-level N to S flowering gradient, OSR flowered 12 days earlier in the South compared to the North. In 2018, drought and heat led to relatively shorter flowering periods. The observed flowering dates were translated into GDD requirements to illustrate how this information can be assimilated in a crop model to benefit in-season crop yield forecasting.

## Author contribution

R.D., M.T., M.Y. and G.L. processed the data. R.D., M.T. and M.V. analyzed the data and wrote the paper. A.C. did the meteo analysis. G.L. provided comments and suggestions on the manuscript.

## Declaration of competing interest

The authors declare that they have no known competing financial interests or personal relationships that could have appeared to influence the work reported in this paper.
