# Supplementary material for: Detecting flowering phenology in oil seed rape parcels with Sentinel-1 and -2 time series
Source: Remote Sens Environ. 2020 Mar 15;239:111660. doi: 10.1016/j.rse.2020.111660 (PMC7043338; doi:10.1016/j.rse.2020.111660)
Supplement: Supplementary file 1 — Supplementary material [file mmc1.pdf]

Supplementary Materials for:

Detecting flowering phenology in oil seed rape parcels with Sentinel-1 and -2 time series

Raphaël d'Andrimont, Matthieu Taymans, Guido Lemoine, Andrej Ceglar, Momchil Yordanov and Marijn van der Velde.

European Commission, Joint Research Centre (JRC), Ispra (VA), Italy

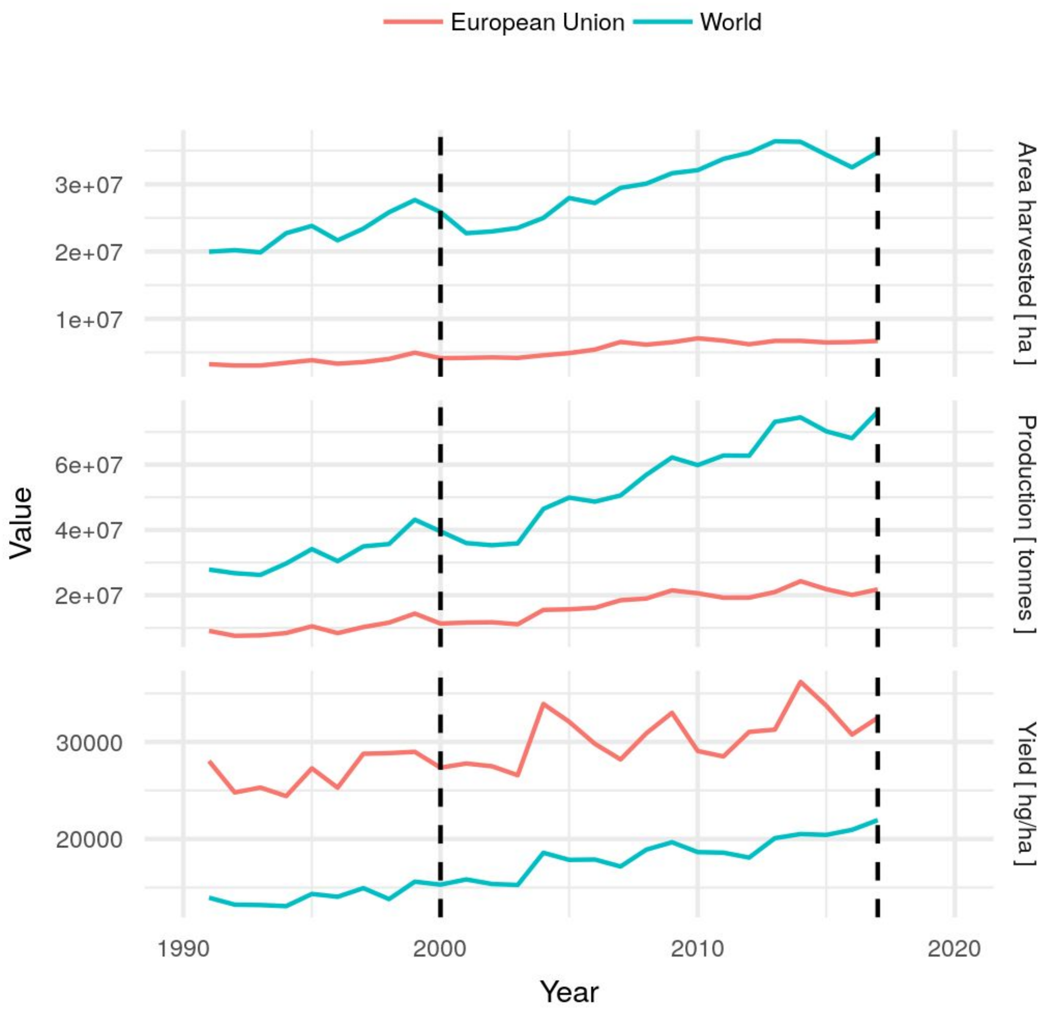

Fig. 1 : Global and European Union rapeseed area harvested, production, and yield (data source : http://www.fao.org/faostat/). Dashed lines correspond to 2000 and 2017.

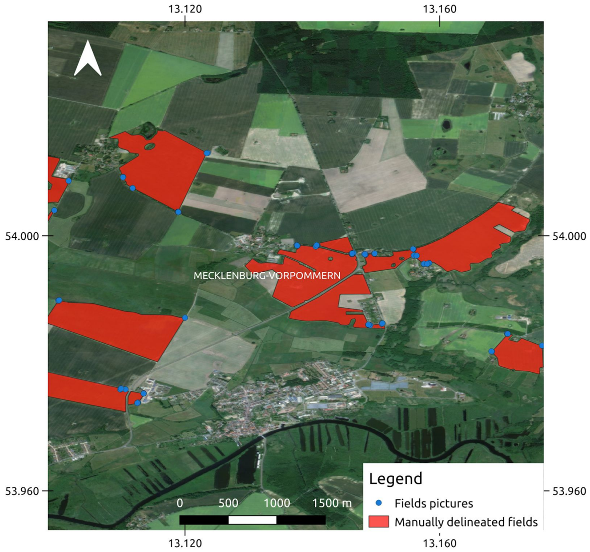

(a)

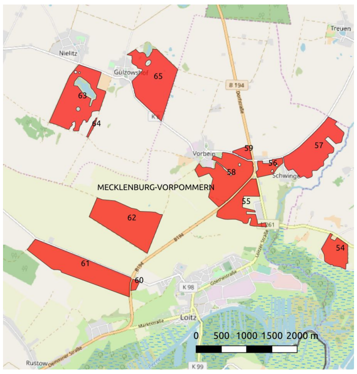

(b)

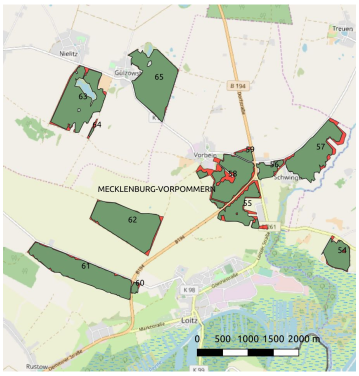

(c)

Fig. 2 : Photo-Interpretation of S2 and high-resolution imagery was used to accurately delineate rapeseed parcels (red polygons) for 229 parcels while classification was used to delineate 32355 parcels automatically (green polygons). For each parcel with in-situ information, pictures were taken from at least two corners (blue points).

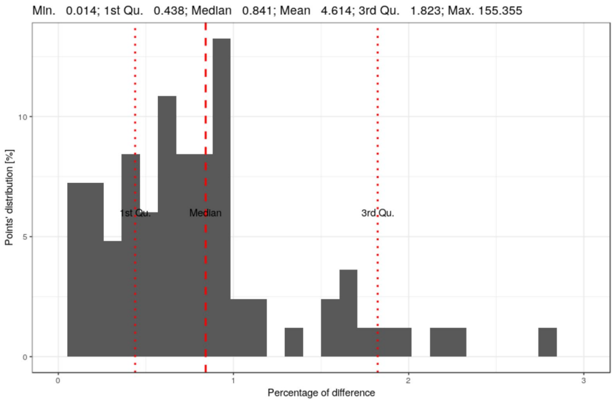

Fig. 3 : Comparison of the individual parcel area of the 229 parcels obtained by visual or classified delineation resulting in a median difference <1% and an average difference <5%. The Percentage difference is expressed in absolute terms.

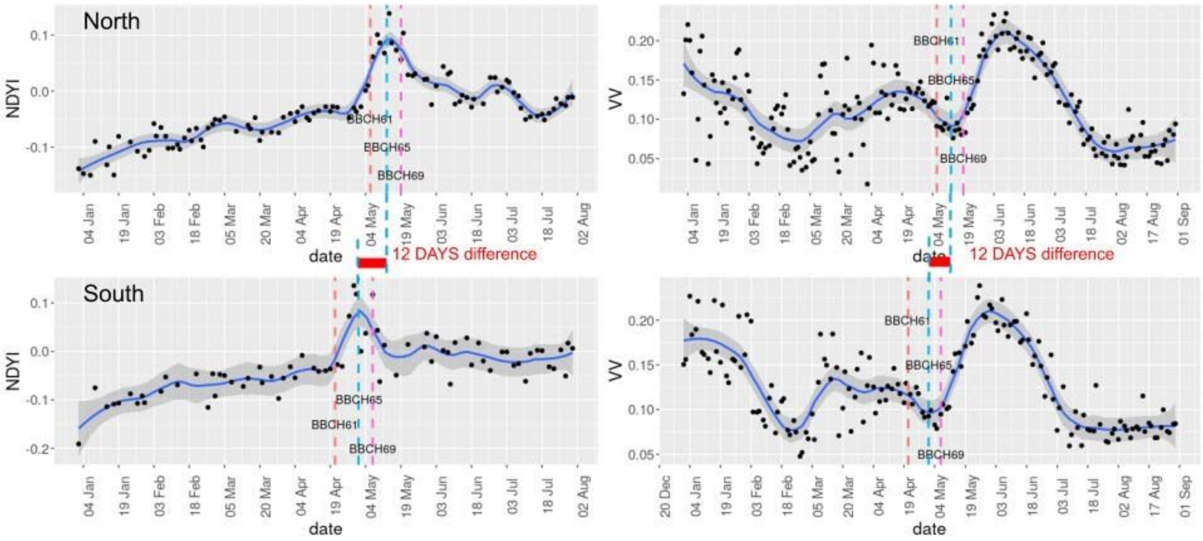

Fig. 4 : Illustration of the 12-day difference measured between the flowering peaks from South and North Germany.

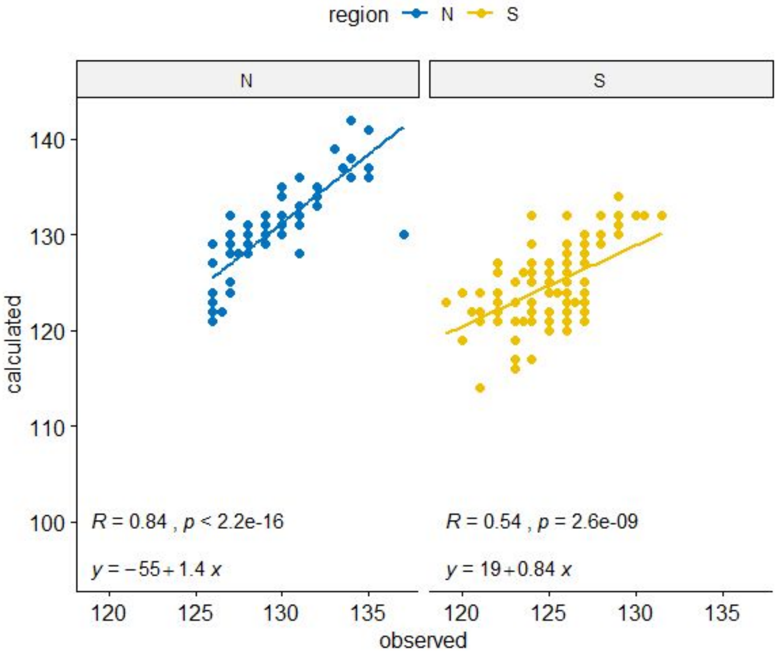

Fig. 5 : Regression between S2 observed DOY of peak flowering and calculated DOY of peak flowering based on GDD for both regions.

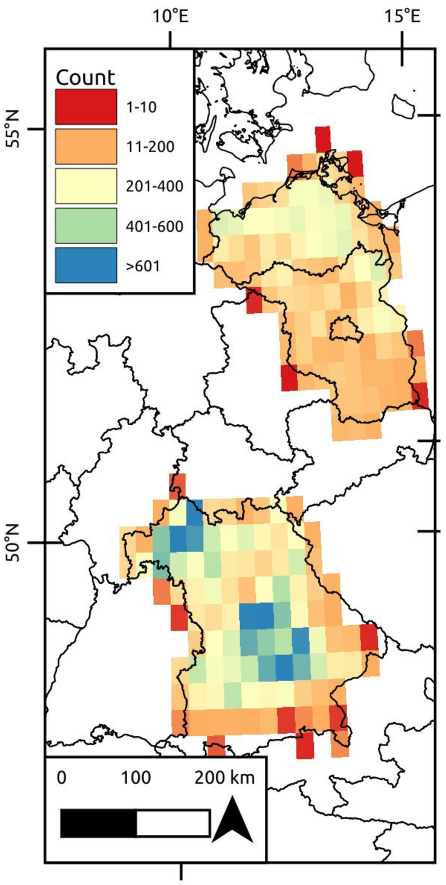

(a)

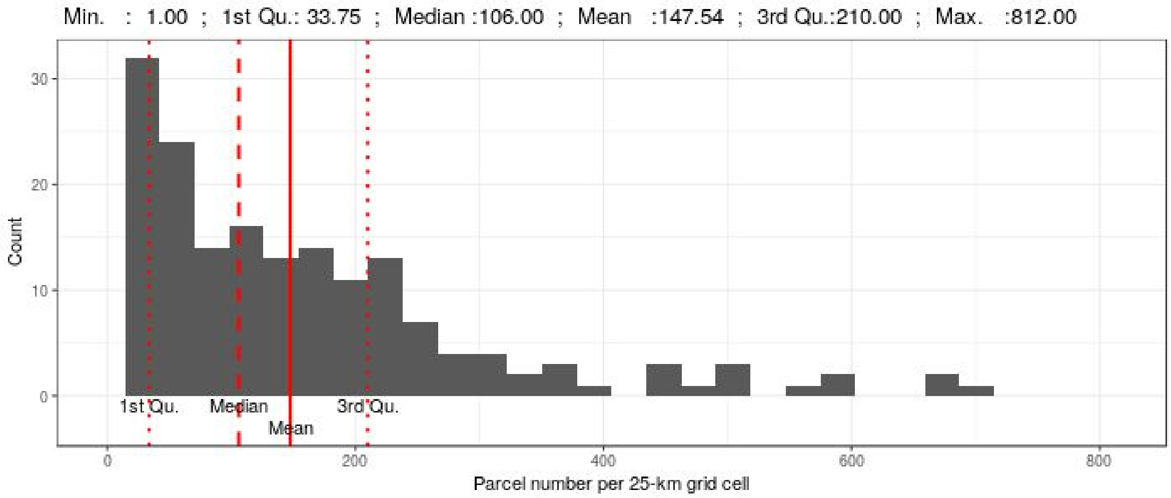

(b)

Fig. 6 : Rapeseed parcel count per 25-km grid cell: (a) shows the spatial distribution on a map while (b) is an histogram showing the distribution of the values.

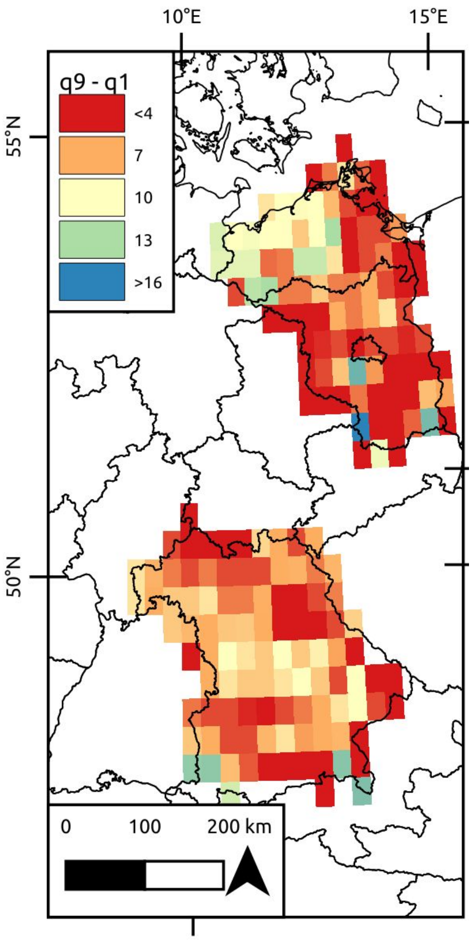

(a)

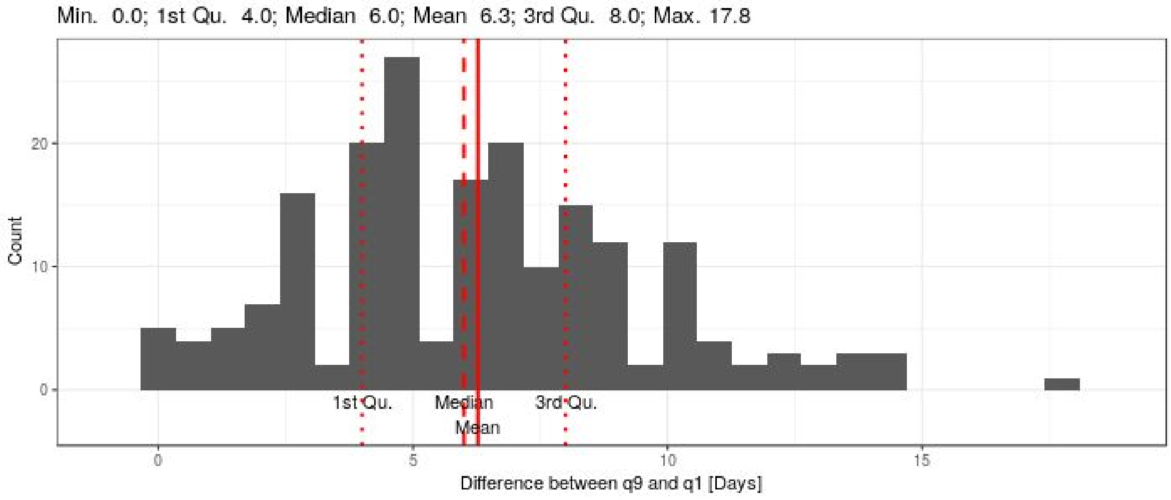

(b)

Fig. 7 : Number of days between q10 and q90 parcel flowering peaks DOY in each 25-km grid cell: (a) shows the spatial distribution on a map while (b) is an histogram showing the distribution of the values

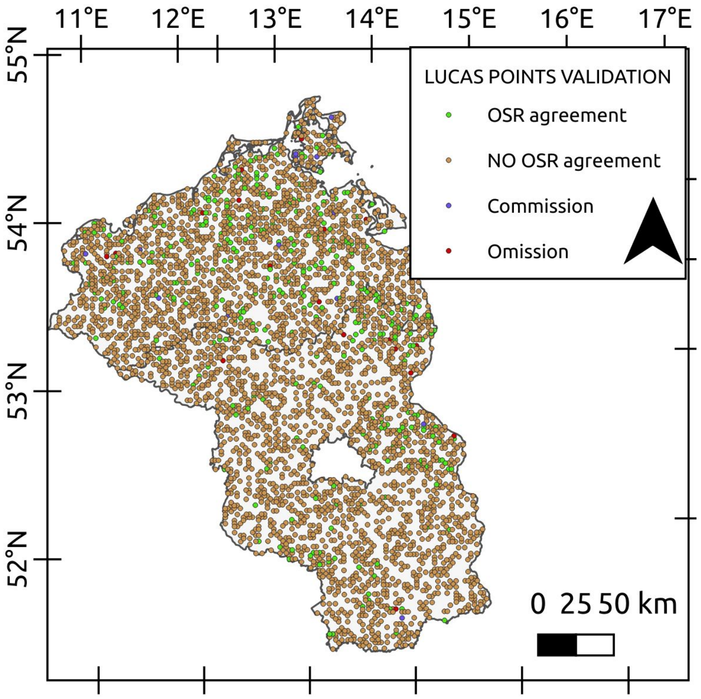

(a)

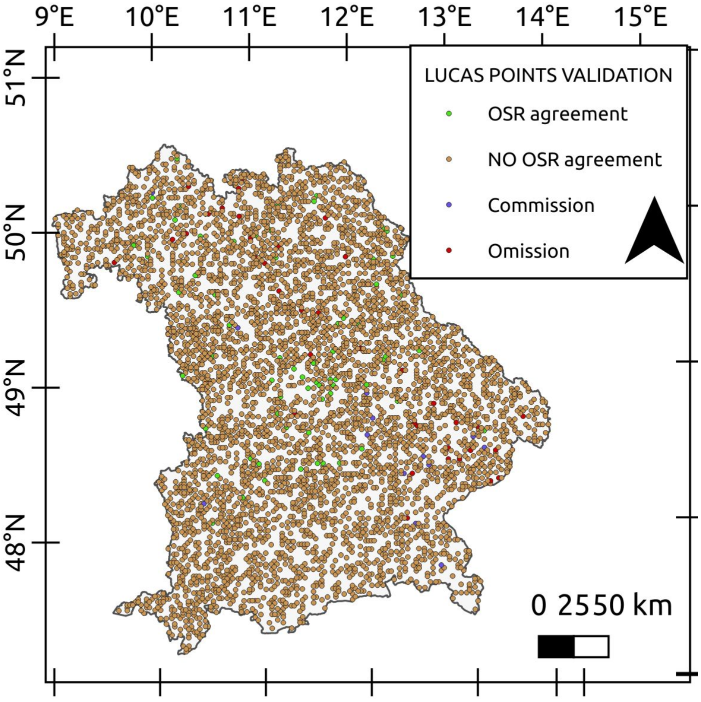

(b)

Fig. 8 : LUCAS micro survey data was collected in 2018 during spring and summer and is therefore perfectly suited validation dataset (3895 points in the N and 4706 points in the S). These maps show the spatial distribution of the validation points. “OSR agreement” and “NO OSR agreement” are correctly classified while “Commission” and “Omission” and incorrectly classified compared to the LUCAS reference data.

Tab. 1a : Confusion matrices resulting of the validation of the classified parcels with LUCAS survey micro data.

NORTH	*LUCAS 2018 REFERENCE*		
	NO OSR	***OSR***	***TOTAL***
NO OSR	*3554*	*18*	*3572*
OSR	*13**	*310*	*323*
TOTAL	*3567*	*328*	*3895*
SOUTH	*LUCAS 2018 REFERENCE*		
	NO OSR	***OSR***	***TOTAL***
NO OSR	*4591*	*37*	*4628*
OSR	*13***	*65*	*78*
TOTAL	*4604*	*102*	*4706*

**Broadleaved woodland (1), Grassland without tree/shrub cover (2), Maize (3), Other artificial areas (1), Other bare soil (5), Rye (1)*

*** Grassland without tree/shrub cover (1), Maize (2),Other artificial areas (1), Other bare soil (1), Other leguminous and mixtures for fodder (1), Other root crops (2), Sugar beet (4), Sunflower (1)*

Tab. 1b : Metrics calculated from the confusion matrices. The Overall Accuracy (OA) ranges from 0.9828 (N) to 0.9845 (S), however, as the OSR class is relatively marginal the OA is not fully relevant. Commission error for OSR ranges from 4.02 % (N) to 16.67 % (S). Omission for OSR ranges from 5.49 % (N) to 36.27 % (S).

	Accuracy	***OSR Commission (%)***	***OSR Omission (%)***	***Sensitivity***	***Specificity***	***Kappa***
N	*0.9920*	*4.02*	*5.49*	*0.9964*	*0.9451*	*0.9480*
S	*0.9894*	*16.67*	*36.27*	*0.9972*	*0.6373*	*0.7169*

Tab. 2 : Area distribution (km²) per land cover in 2018 for both study sites (N and S) obtained from Corine Land Cover 2018.

CLC code	***Label***	***N (Area km²)***	***N (Area %)***	***S (Area km²)***	***S (Area %)***
111	*Continuous urban fabric*	6	0.0001	29	0.0004
112	*Discontinuous urban fabric*	2101	0.0399	3722	0.0529
121	*Industrial or commercial units*	468	0.0089	673	0.0096
122	*Road and rail networks and associated land*	12	0.0002	30	0.0004
124	*Airports*	77	0.0015	62	0.0009
131	*Mineral extraction sites*	147	0.0028	65	0.0009
132	*Dump sites*	14	0.0003	5	0.0001
133	*Construction sites*	5	0.0001	2	0.0000
141	*Green urban areas*	82	0.0016	76	0.0011
142	*Sport and leisure facilities*	216	0.0041	188	0.0027
211	*Non-irrigated arable land*	22837	0.4337	23556	0.3349
222	*Fruit trees and berry plantations*	56	0.0011	140	0.0020
231	*Pastures*	8055	0.1530	14519	0.2064
242	*Complex cultivation patterns*	5	0.0001	164	0.0023
243	*Land principally occupied by agriculture, with significant areas of natural vegetation*	80	0.0015	126	0.0018
311	*Broad-leaved forest*	3246	0.0616	3845	0.0547
312	*Coniferous forest*	11896	0.2259	14474	0.2058
313	*Mixed forest*	955	0.0181	6582	0.0936
321	*Natural grasslands*	249	0.0047	652	0.0093
322	*Moors and heathland*	246	0.0047	204	0.0029
324	*Transitional woodland-shrub*	275	0.0052	348	0.0049
331	*Beaches, dunes, sands*	35	0.0007	27	0.0004
333	*Sparsely vegetated areas*	3	0.0001	68	0.0010
411	*Inland marshes*	230	0.0044	17	0.0002
412	*Peat bogs*	43	0.0008	128	0.0018
511	*Water courses*	67	0.0013	145	0.0021
512	*Water bodies*	1253	0.0238	498	0.0071
	TOTAL	**52659**	**1**	**70345**	**1**
